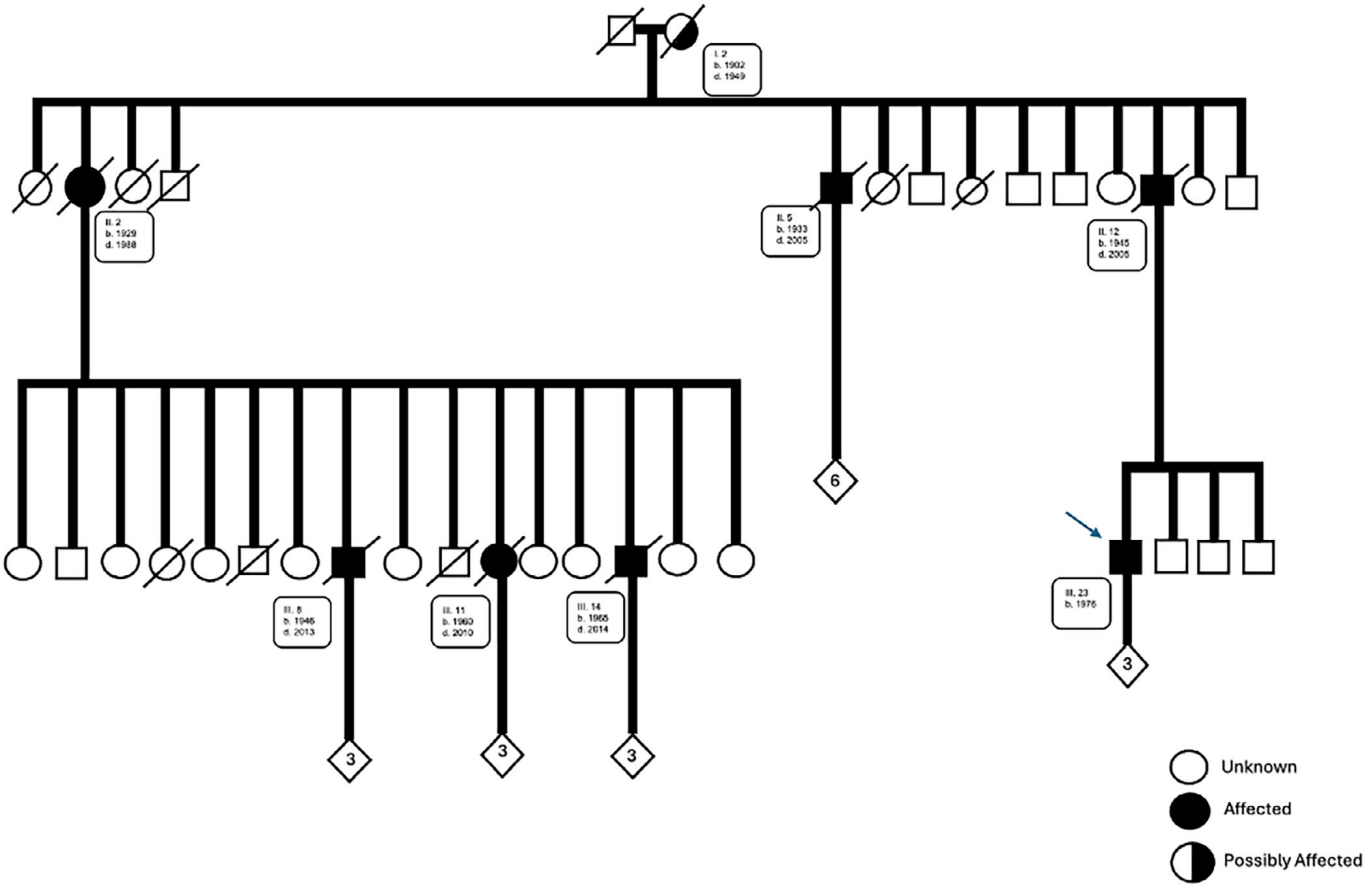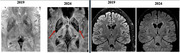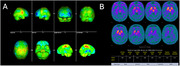# Fatal familial insomnia with a Lewy‐like presentation

**DOI:** 10.1002/alz70857_102926

**Published:** 2025-12-25

**Authors:** Mauricio Silva Teixeira, Anna Maria Gomes, Hindalis Ballesteros Epifanio, Cícero José Nunes Vaz, Patricia Faria Scherer, Sonia Maria Dozzi Brucki, Edson Bor‐Seng‐Shu

**Affiliations:** ^1^ Hospital das Clinicas HCFMUSP, Faculdade de Medicina, Universidade de Sao Paulo, Sao Paulo, Sao Paulo, Brazil; ^2^ Hospital Israelita Albert Einstein, Sao Paulo, Sao Paulo, Brazil

## Abstract

**Background:**

Fatal Familial Insomnia (FFI) is a rare autosomal dominant prion disease. Herein, we present a case of a patient who was diagnosed with FFI, with symptoms initially resembling dementia with Lewy bodies (DLB).

**Method:**

A 48‐year‐old Brazilian male with a six‐year history of insomnia presented with rapidly progressive dementia with predominant amnestic, dysexecutive, and visuospatial impairments; marked cognitive fluctuations; complex visual hallucinations; delusions; parkinsonism; severe dysautonomia; neuroleptic hypersensitivity; REM sleep behavior disorder; agrypnia excitata; and cerebellar ataxia. Several family members exhibited similar symptoms in an autosomal dominant pattern (Figure 1). Serum workup was unremarkable. CSF analysis was normal, except for 14‐3‐3 protein, which was elevated (3439 AU/ml). Tau protein was low and RT‐QuIC was negative. Brain MRI showed cortical, basal nuclei and thalamic atrophy compared to the previous image from 2019 (Figure 2). FDG‐PET indicated glycolytic metabolism deficit predominantly in the left frontal and parietal regions (Figure 3A), and TRODAT SPECT showed a bilateral reduction in dopamine transporter availability, especially in the left striatum and putamen (Figure 3B). Polysomnography revealed severe altered sleep architecture, reduced REM sleep, and increased microarousals and abnormal motor activity during sleep. Genetic testing was performed, and a pathogenic variant c.532G>A:p (Asp178Asn) in heterozygosis in exon 2 was found in the prion protein (PRNP) gene.

**Result:**

FFI is caused by a missense mutation in the PRNP gene involving a substitution of aspartic acid (D) with asparagine (N) at codon 178, known as the D178N mutation. The FFI phenotypes are strongly linked to the methionine (Met) / valine (Val) codon 129 polymorphism on the same allele as the D178N mutation. In our case, although glycolytic hypometabolism on the thalamus had not been seen, there was a marked striatum involvement in TRODAT SPECT, in congruence with his parkinsonism. Rare cases of FFI were reported in Latin American countries, and we have found in the literature only one case report regarding FFI that mimicked DLB symptoms.

**Conclusion:**

FFI can present with a heterogeneous phenotype. Patients with symptoms resembling DLB but showing alarm signs (rapid progressive dementia, multiple sleep abnormalities, strong family history) should prompt a more thorough investigation.